# A comparison of iCare and Goldmann applanation tonometry measurements during the COVID-19 pandemic: a retrospective study

**DOI:** 10.1007/s10792-024-03220-8

**Published:** 2024-06-23

**Authors:** Shreya Swaminathan, Amber M. Kisielewski, M. Hossein Nowroozzadeh, Shahin Hallaj, Daniel Lee, Natasha N. Kolomeyer, Jonathan S. Myers, Reza Razeghinejad

**Affiliations:** 1 Sidney Kimmel Medical College, Thomas Jefferson University, Philadelphia, PA 19107 USA; 2 Glaucoma Service, Wills Eye Hospital, 840 Walnut St, Philadelphia, PA 19107 USA; 3 Department of Ophthalmology, Shiraz University of Medical Sciences, Shiraz, Iran

**Keywords:** Goldmann applanation tonometry, Intraocular pressure, Glaucoma, iCare

## Abstract

**Purpose:**

To evaluate factors associated with differences in intraocular pressure (IOP) readings between iCare and Goldmann applanation tonometry (GAT) in established glaucoma patients.

**Methods:**

This retrospective comparative study included clinical data of 350 eyes from 350 established glaucoma patients who had iCare and GAT IOP measured by an ophthalmic technician and a glaucoma specialist, respectively. The main outcome measure was the difference in IOP measurements of the right eyes with iCare and GAT.

**Results:**

The intraclass correlation coefficient (ICC) between GAT and iCare was 0.90. The mean IOP difference between tonometers was − 0.18 ± 2.89 mmHg. Bland–Altman plots indicated a 95% limit of agreement of − 5.8 to 5.5 mmHg. Central corneal thickness (CCT) and age were significantly correlated with the difference in IOPs of the iCare and GAT. GAT-IOP and age were significantly associated with the absolute difference in measured IOP of the two tonometers. The difference in measurements was not significantly associated with prior glaucoma surgery, average global index of optical coherence tomography, axial length, technician years of experience and certification, and IOP range.

**Conclusion:**

Although there is good agreement between the iCare and GAT mean values, these devices are not interchangeable in glaucoma patients due to the wide range of the limit of agreement.

## Introduction

Intraocular pressure (IOP) is the only modifiable risk factor to decelerate glaucomatous optic neuropathy and progressive visual field loss. Therefore, accurate IOP monitoring is essential in glaucoma management [1]. Goldmann applanation tonometry (GAT) is considered the gold standard tonometer. However, GAT requires significant training and the use of a slit lamp. Additionally, a topical anesthetic with fluorescein dye must be applied to the eye, which may cause irritation and reflex blepharospasm [2]. The iCare is a handheld tonometer that has gained significant interest among clinicians due to its portability, usability, and ability to measure IOP without the use of a topical anesthetic [3].

The coronavirus disease 2019 (COVID-19) pandemic caused practitioners to re-evaluate the most appropriate instrument for IOP measurement. The diameter and surface area of iCare probes are approximately 1.7 mm and 4.6 mm^2^, respectively, as compared with the GAT tip, which has a diameter of 3.06 mm and a surface area of 7.35 mm^2^ [4, 5]. A smaller contact surface may reduce the risk for infection. Additionally, the iCare probe tips are disposable, suggesting that iCare may have the potential to decrease virus transmission. There are disposable GAT tips available. However, in a 2020 survey of members of the American Optometry Association (AOA) and the American Glaucoma Society (AGS), survey results suggest that 165 out of 276 survey participants use reusable tips, suggesting that there is a large portion of providers that clean and re-use GAT tips [6]. In contrast, iCare probe tips are not meant to be reused.

Multiple studies have highlighted high inter-device correlation between GAT and iCare tonometry [2, 7, 8]. Higher IOP and central corneal thickness (CCT) may impact the agreement level between iCare and GAT recorded IOP [2, 7, 9–12]. It has also been shown that iCare readings are likely not influenced by factors such as spherical equivalent and axial length [8, 13, 14]. The purpose of this study was to evaluate the agreement between GAT and iCare tonometry and the factors associated with their readings in a larger and more diverse population of glaucoma patients.

## Materials and methods

This retrospective cohort study was approved by the Institutional Review Board at Wills Eye Hospital. The research was conducted by the Health Insurance Portability and Accountability Act of 1996 and adhered to the tenets of the Declaration of Helsinki. The statistical analysis was conducted on right eyes of patients seen by the Wills Eye Glaucoma Service between March 1, 2020 and March 31, 2021, although all measurements for both eyes were obtained. IOP was measured with the iCare by ophthalmic technicians and with the GAT afterwards by a trained glaucoma specialist (RR). These readings were conducted between 1 to 77 min from each other. Exclusion criteria included age < 17 years, astigmatism greater than 3 diopters, corneal opacities and infection, and first postoperative month.

Demographic data, visual acuity (VA), IOP, axial length (AL), central corneal thickness (CCT), visual field indices, and mean retinal nerve fiber layer thickness of optical coherence tomography were collected through retrospective chart review. Glaucoma severity was assessed using the Hodapp-Parrish-Anderson Criteria [15].

Although data of both eyes were collected, the right eye was randomly chosen for analysis to decrease bias and improve validity. The analysis of the left eyes’ data was used to test the reproducibility of the findings.

The primary outcome measures were the agreement between iCare and GAT IOP readings and the factors influencing the differences between the two devices. IBM SPSS Statistics software version 26 (SPSS Inc., Chicago, IL) and MedCalc version 12.2.1 (MedCalc Software, Mariakerke, Belgium) were used to conduct statistical analyses. The data were reported as mean ± standard deviation (SD). Independent sample t-tests were used to investigate the relationship of the mean difference in IOPs ≤ 21 and > 21 mmHg. The chi-square test was used to investigate the distribution of the difference in IOP measurements ≤ 21 mmHg and > 21 mmHg. The intraclass correlation coefficient (ICC) and Bland–Altman plots were used to determine the agreement between the tonometers. Univariable linear regression models were used to evaluate the factors affecting the differences between tonometers. Variables with a *P*-value < 0.1 were evaluated with multivariable regression models. A *P* value less than 0.05 was considered as significant.

## Results

**Fig. 1:**
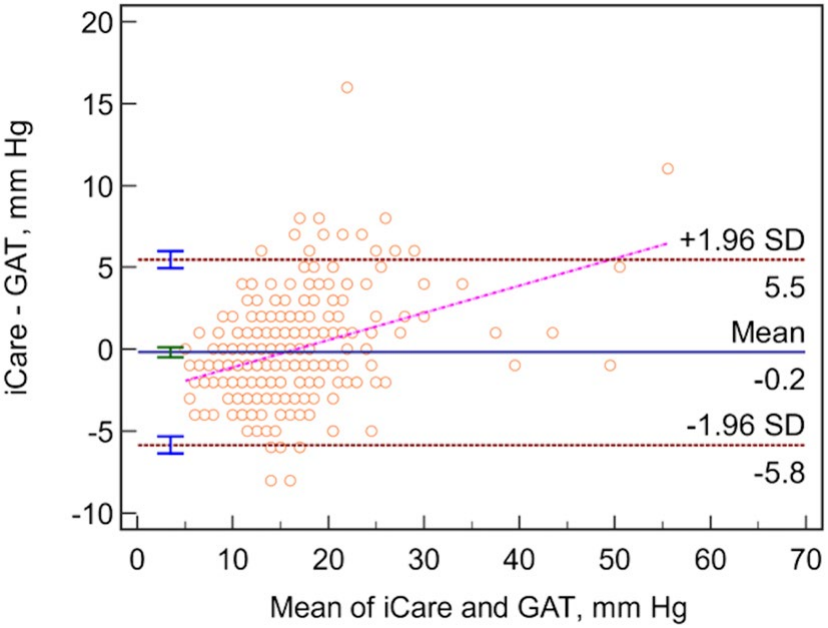
Bland–Altman plot depicting relationship between mean IOP and the iCare-GAT IOP difference

**Fig. 2:**
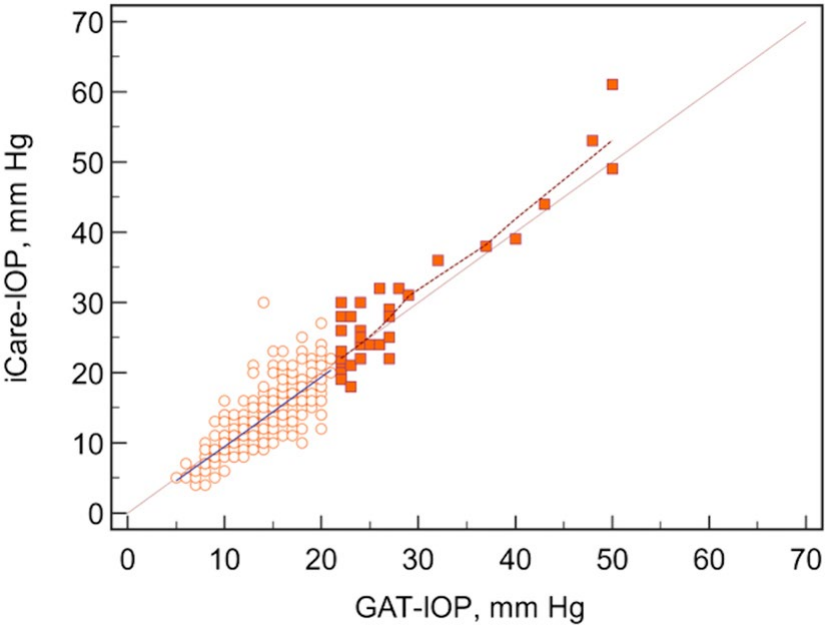
Plot depicting the relationship between IOP measured by GAT and iCare at IOP ≤ 21 mmHg (open circle) and at IOP > 21 mmHg (closed square)

This study included 350 right eyes of 350 patients. Clinical characteristics and demographic data are shown in Table 1. The mean difference in IOP measurements between the two devices (Δ iCare-GAT) was − 0.18 ± 2.89 mmHg with a 95% limit of agreement of − 5.8 to 5.5 mmHg on a Bland–Altman plot (Fig. 1). There was a high correlation between the two methods of IOP measurement, as seen in Fig. 2, with an overall intraclass correlation coefficient (ICC) of 0.90. There was no significant difference in the average Δ iCare-GAT between eyes with IOP ≤ 21 mmHg and IOP > 21 mmHg (Table 2).

**Table 1:** Baseline characteristics of participants

Age in years, Mean ± SD	65.2 ± 14.7
Female, N (%)	181 (51.7%)
*Type of glaucoma*	
POAG, N (%)	232 (66.3%)
PACG, N (%)	50 (14.3%)
Secondary, N (%)	68 (19.4%)
*Glaucoma severity*	
Mild glaucoma, N (%)	152 (46.6%)
Moderate glaucoma, N (%)	79 (24.2%)
Severe glaucoma, N (%)	95 (29.1%)
*Prior surgeries, N (%)*	74 (21.1%)
Trabeculectomies, N (%)	36 (10.3%)
Tube shunts, N (%)	34 (9.7%)
Xen, N (%)	3 (0.9%)
MIGS, N (%)	12 (3.4%)
PI, N (%)	41 (11.7%)
Laser trabeculoplasty, N (%)	66 (18.9%)
CPC, N (%)	13 (3.7%)
*Lens status*	
Aphakic, N (%)	8 (2.3%)
Pseudophakic, N (%)	141 (40.3%)
Phakic, N (%)	201 (57.4%)
LogMAR VA, Mean ± SD	0.41 ± 0.67
*IOP*	
GAT IOP, mmHg Mean ± SD	15.6 ± 5.9
iCare IOP, mmHg Mean ± SD	15.4 ± 6.9
CCT, µm, Mean ± SD	551 ± 48
AL, mm, Mean ± SD	24.13 ± 1.76
OCT Global index, µm, Mean ± SD	77 ± 22
Visual field Mean deviation (Mean ± SD)	8.4 ± 7.6
Time between IOP readings, minutes, Mean ± SD	17.5 ± 12.4

AL: axial length; ALT: argon laser trabeculoplasty; CPC: cyclophotocoagulation; CCT: central corneal thickness; GAT: Goldmann applanation tonometry; IOP: intraocular pressure; MIGS: minimally invasive glaucoma surgery; PACG: primary angle-closure glaucoma; PI: laser peripheral iridotomy; POAG: primary open-angle glaucoma; SE: spherical equivalent; SLT: selective laser trabeculoplasty; Trab: trabeculectomy; VA: visual acuity

**Table 2:** Comparison of IOP measurements with the Goldmann applanation and iCare tonometers in right eyes

	Total (n = 350)	Subgroup analysis		
		IOP ≤ 21 mmHg (n = 313)	IOP > 21 mmHg (n = 37)	*P*-value
Mean difference, mm Hg	− 0.18 ± 2.89	− 0.31 ± 2.75	0.92 ± 3.72	0.059*
*P*-value^†^	0.244	0.047	0.142	
ICC (95% CI)	0.899 (0.877–0.918)	0.755 (0.703–0.799)	0.913 (0.839–0.954)	
95% LoA, mm Hg	− 5.8 to 5.5	− 5.7 to 5.1	− 6.4 to 8.2	
Difference > 2 mm Hg (%)	31.1%	30.0%	40.5%	0.192^‡^
Difference > 3 mm Hg (%)	20.3%	18.5%	35.1%	0.018^‡^

*Independent-sample *T*-test; †Paired-sample *T*-test; ‡Chi-Squared test; CI: confidence interval; ICC: intraclass correlation coefficient; IOP: intraocular pressure; LoA: limits of agreement

Δ iCare-GAT was significantly correlated with age, GAT-IOP, and CCT in univariable analysis (Table 3). No significant association was found between Δ iCare-GAT and sex, visual acuity, AL, optical coherence tomography average nerve fiber layer thickness, lens status, glaucoma severity and type, prior surgery, and the qualification and experience of the technicians taking the iCare measurement.

**Table 3:** Factors affecting IOP measurement difference between iCare and GAT (iCare—GAT) in right eyes

Factors	Univariable analysis		Multivariable analysis	
	Coefficient (95% CI)	*P*-value	Coefficient (95% CI)	*P*-value
Age	− 0.029 (− 0.049 to − 0.008)	0.006	− 0.039 (− 0.064 to − 0.013)	0.003
GAT IOP	0.068 (0.017–0.119)	0.009		0.892
CCT	0.011 (0.003–0.018)	0.005	0.008 (0.001–0.016)	0.027
Time between IOP readings	0.024 (− 0.001 to 0.048)	0.056		0.794
Lens status (aphakic vs. others)	1.979 (− 0.046 to 4.003)	0.055		0.908

CCT, central corneal thickness; CI, confidence interval; GAT, Goldmann applanation tonometry; IOP, intraocular pressure

The absolute value of Δ iCare-GAT was significantly correlated with age, GAT-IOP, and lens status (aphakic vs. others) in univariable analysis (Table 4). No significant association was found between the absolute value of Δ iCare-GAT and sex, visual acuity, AL, CCT, optical coherence tomography average nerve fiber layer thickness, lens status (pseudophakic vs others), glaucoma severity, glaucoma type, prior surgery, and the qualification and experience of the technician taking the iCare measurement.

**Table 4:** Factors affecting the absolute difference in IOP measurement between iCare and GAT in the right eyes

Factors	Univariable analysis		Multivariable analysis	
	Coefficient (95% CI)	*P*-value	Coefficient (95% CI)	*P*-value
Age	− 0.025 (− 0.039 to − 0.011)	< 0.001	− 0.021 (− 0.037 to − 0.005)	0.012
GAT IOP	0.078 (0.044–0.113)	< 0.001	0.082 (0.040–0.124)	< 0.001
Time between IOP readings	0.014 (− 0.002 to 0.031)	0.089		0.191
Lens status (Pseudophakic vs. others)	− 0.404 (− 0.825 to 0.016)	0.060		0.595
Lens status (aphakic vs. others)	1.668 (0.293–3.043)	0.018		0.109

CCT, central corneal thickness; CI, confidence interval; GAT, Goldmann applanation tonometry; IOP, intraocular pressure

## Discussion

iCare tonometry has gained popularity during the COVID-19 pandemic due to its portability, lack of need for anesthetic agents, disposable probes, and minimal eye surface contact [16, 17]. To evaluate iCare accuracy in the real world, we compared iCare tonometry with GAT in glaucoma patients to determine the factors that may influence IOP measurements.

The findings of this study suggest that iCare and GAT IOP readings have high levels of correlation which is in accordance with some of the prior studies [7, 18]. ICC in our study was 0.90 between the two devices, slightly lower than what was reported by Pakrou et al. (0.93 and 0.95) for left and right eyes, respectively [2]. This may be attributable to the larger sample size and inclusion of only glaucomatous in our study. Additionally, in our study a single ophthalmologist did all GAT measurements, and in Pakrou et al.’s study the measurements were done by two examiners. The ICC value in our study was comparable to that of Munkwitz et al. (0.87); notably our study was conducted in a larger cohort [9].

Although there was no significant difference in the average measurements between the two devices, a 95% limit of agreement between − 5.8 to 5.5 mmHg was observed which suggests that iCare tonometry and GAT may not be interchangeable. This limit of agreement is comparable to what was reported by Kim et al. for the iCare PRO (− 4.52 to 8.37 mmHg) [13]. Fernandes et al. [19] reported a narrower 95% limit of agreement between iCare and GAT (± 3.98 mmHg) and had a greater number of measurements with IOP differences less than 3 mmHg (82.6%). This may be attributable to the inclusion of eyes without glaucoma, whereas our study only included glaucomatous eyes. In another study on non-glaucomatous eyes, Kato et al. [8] reported a narrower 95% limit of agreement and a mean difference between GAT and iCare IOP measurements of 2.46 ± 2.10 mmHg, which may suggest that iCare may be more accurate for non-glaucomatous eyes within normal IOP limits. Munkwitz et al. [9] also reported a wider 95% limit of agreement (− 8.67 to 10.25 mmHg) than our study, which may be because of the larger range of IOP measurements and a greater mean GAT- IOP (20.80 ± 9.38 mmHg) and iCare IOP (21.59 ± 9.17 mmHg).

Age was significantly correlated with the difference in IOPs between iCare and GAT. There was a 0.4 mmHg lower IOP reading by iCare compared with GAT for each decade increase in age. Age was also shown to be significantly correlated with the absolute difference in IOP measurements. With each decade increase, there was 0.2 mmHg less absolute difference in IOP reading. This suggests that younger age is associated with a slightly greater absolute difference in IOP measurements between iCare and GAT. González-Méijome et al. and Nakakura et al. reported lower IOP measurements using iCare with increases in age [20, 21]. This may be due to age-related corneal biomechanical changes. Grabner et al. [22] reported that corneal resistance to indentation was negatively correlated with age, suggesting that older patients may have decreased corneal rigidity. iCare tonometry utilizes the principle of rebound tonometry wherein a moving probe collides with the eye and the motion parameters of the probe are monitored and used for IOP calculation [1]. Therefore it is possible that age-related corneal biomechanical changes may account for differences in IOP measurements. This negative correlation between differences in IOP and age has been reported with iCare HOME and iCare ONE [23].

CCT was significantly correlated with the difference in IOP measurement between iCare and GAT. Previous studies have reported that GAT is most accurate with a CCT of 520 μm and may over- and underestimate IOP in thicker and thinner corneas, respectively [24, 25]. However, CCT has also been shown to influence rebound tonometers like iCare [26, 27]. Our study had indicated that CCT had a greater average effect on iCare measurements. For every 125 µm increase in CCT, there was a 1 mmHg increase in the difference between iCare and GAT IOP measurements. This was similar to the 100 µm increase in CCT per 1 mmHg difference in IOP readings reported by Pakrou et al. [2]. However, our study reported a lesser influence of CCT on difference in IOP measurements as compared to Brusini et al. [1] who reported a CCT change of 10 μm per 0.7 mmHg deviation in iCare, which may be due to their lower sample size compared to ours.

GAT-IOP was significantly associated with the absolute difference in IOP between iCare and GAT. Each 1 mmHg increase in GAT-IOP resulted in a 0.8 mmHg absolute difference in IOP readings between iCare and GAT. This suggests that higher IOP was associated with a greater absolute difference of iCare and GAT measurements. There was also a significantly greater number of patients with an IOP difference greater than 3 mmHg between the two tonometers in the group of patients with IOP > 21 mmHg than the group with IOP ≤ 21 mmHg. Additionally, 40.5% of patients with an IOP > 21 had a difference in readings > 2 mmHg, whereas only 30.0% of patients in the group with an IOP ≤ 21 had a difference in readings between the two tonometers of > 2 mmHg. Although this was not statistically significant, this finding seems to be clinically significant, indicating more discrepancies at higher IOPs. The iCare tended to overestimate IOP at higher IOP ranges which is similar to what was reported by Nakamura et al. [10].

Prior study has investigated GAT interrater reproducibility and reports a 95% limit of agreement of − 1.4 to 3.1 and − 2.2 to 3.5 mmHg [28]. This is a narrower limit of agreement than ours comparing GAT and iCare measurements, and may suggest that there is more variability in repeated IOP measurements with iCare and GAT than with the GAT alone.

Axial length was not associated with an IOP difference between iCare and GAT, which agrees with Kim et al.’s study [13]. Ophthalmic technician qualification and years of training were also not associated with the difference in IOP measurement between iCare and GAT. Abraham et al. [29] has shown that iCare IOP measurements have been shown to be comparable to those of GAT even when iCare is taken by inexperienced tonometrists, substantiating the claim of iCare’s usability.

This study has several limitations including its retrospective nature. However the large sample size, the inclusion of glaucoma patients with varying disease severity and IOPs, the exclusion of those with high astigmatism, and the performance of statistical analysis on only one eye strengthen the validity of the findings. The biomechanical properties of the cornea such as corneal hysteresis and corneal curvature were not evaluated and may be addressed in future studies.

In conclusion, there was good agreement between the iCare and GAT mean values, but they are not interchangeable when measuring the IOP in glaucoma patients because of the wide range of the limit of agreement. CCT and age were significantly associated with increased differences in measurements.
